# Laser-induced transformation of supramolecular complexes: approach to controlled formation of hybrid multi-yolk-shell Au-Ag*@*a-C:H nanostructures

**DOI:** 10.1038/srep12027

**Published:** 2015-07-08

**Authors:** A. A. Manshina, E. V. Grachova, A. V. Povolotskiy, A. V. Povolotckaia, Y. V. Petrov, I. O. Koshevoy, A. A. Makarova, D. V. Vyalikh, S. P. Tunik

**Affiliations:** 1 Institute of Chemistry, St. Petersburg State University, Universitetskii pr. 26, St. Petersburg, 198504, Russia; 2 Center for optical and laser materials research, Research park, St. Petersburg State University, Ulianovskaya St. 5, St. Petersburg, 198504, Russia; 3 Interdisciplinary Resource Center for Nanotechnology, St. Petersburg State University, Ulianovskaya St. 1, St. Petersburg, 198504, Russia; 4 University of Eastern Finland, Joensuu, 80101, Finland; 5 Institut für Festkörperphysik, Technische Universität Dresden, D-01062 Dresden, Germany

## Abstract

In the present work an efficient approach of the controlled formation of hybrid Au–Ag–C nanostructures based on laser-induced transformation of organometallic supramolecular cluster compound is suggested. Herein the one-step process of the laser-induced synthesis of hybrid multi-yolk-shell Au-Ag@a-C:H nanoparticles which are bimetallic gold-silver subnanoclusters dispersed in nanospheres of amorphous hydrogenated a-C:H carbon is reported in details. It has been demonstrated that variation of the experimental parameters such as type of the organometallic precursor, solvent, deposition geometry and duration of laser irradiation allows directed control of nanoparticles’ dimension and morphology. The mechanism of Au-Ag@a-C:H nanoparticles formation is suggested: the photo-excitation of the precursor molecule through metal-to-ligand charge transfer followed by rupture of metallophilic bonds, transformation of the cluster core including red-ox intramolecular reaction and aggregation of heterometallic species that results in the hybrid metal/carbon nanoparticles with multi-yolk-shell architecture formation. It has been found that the nanoparticles obtained can be efficiently used for the Surface-Enhanced Raman Spectroscopy label-free detection of human serum albumin in low concentration solution.

Hybrid metal/dielectric or metal/semiconductor nanostructures are considered to be a new class of multifunctional nanomaterials that has promising properties for sensing technologies and diagnostics in medicine, biology, ecology as well as in catalysis^1^, photocatalysis^2^,^3^, analytical chemistry^4^, optical applications as systems with enhanced third-order electronic susceptibility^5^. The hybrid nanostructures of this sort demonstrate wide variety of specific characteristics, which can be tuned by modification of morphology and composition of their components. Hybrid nanostructures are typically based on such carriers as oxides (TiO_2_, ZrO_2_, SiO_2_, ZnO)^6^,^7^,^8^, polymers^9^ and contain homometalic (Ag, Au, Cu, Pt, etc.)^10^ or heterometallic nanoparticles dispersed in or spread onto the surface of the nanostructures of various morphology – nanoparticles, nanotubes, nanoflakes, etc^11^,^12^,^13^.

In the past decade a special attention was focused on hybrid materials based on various allotropes of carbon that is considered to be the 21st century material and mono/bi-metal nanoparticles with remarkable plasmonic properties. Carbon/metal hybrids are regarded as a new family of multifunctional nanomaterials which are highly promising for multifarious applications thanks to enhanced characteristics resulting from synergetic effect of components, mutual influence of their electronic structures, their physical and chemical interaction. The main impact of nanometal phase of such hybrid materials is typically governed by localized surface-plasmon resonance (SPR) to give surface-enhanced Raman scattering (SERS) and metal enhanced fluorescence (MEF). Silver nanoparticles show the largest effect in these phenomena but gold is also considered as a prospective component due to chemical stability and compatibility with various target materials (especially biological), that stimulates growing attention to bimetallic Au-Ag nano-aggregates and associated expansion of the application areas. The carbon phase functionality is determined by its electronic structure related to both allotropic form and morphology. These observations clearly point to various ways of bimetallic (in particular Au-Ag)/carbon hybrid nanomaterials applications, which can cover a wide range of technologies: such as analysis (electrochemical, SERS, MEF), catalysis and production of building blocks for functional devices, e.g. photonic crystals^14^,^15^,^16^.

By the present time considerable number of approaches have been developed for the hybrid nanostructures’ (particularly carbon/metal hybrids) fabrication, including atom beam sputtering, “*in situ*” nanoparticles (NPs) formation in an ongoing modified Stober reaction, doping of pre-prepared nanospheres with metal NPs, “layer-by-layer” coating, etc^17^,^18^,^19^. All these methods are rather complex and multistage, and typically based on a separated synthesis of precursors – metal nanoparticles or nanoporous carbon nanoparticles – followed by sophisticated chemistry, which makes possible deposition of the metal NPs on the surface of the nanoscale dielectric spheres or embedding them into the spheres. Because of complicated technology the resulted hybrid nanostructures often display non-uniform distributions, incomplete coverage, poorly controlled composition, lack of repeatability, poor long-term stability.

Here we suggest alternative efficient approach based on laser-induced transformation of organometallic supramolecular precursors that results in controlled formation of the hybrid C-Au-Ag nanostructures. The precursors belong to the family of supramolecular complexes and consist of hetero-metallic cluster core accommodated into carbon-rich ligand environment. We found that such precursors favor formation of hybrid nanostructures with precise composition, while the characteristics of laser irradiation define their structure and morphology. The photochemical instability of the supramolecular complex allows for its decomposition under mild illumination conditions thus avoiding side effects of thermal phenomena (delocalization of the deposition process, thermal decomposition of the deposits, etc.). Earlier we demonstrated availability of laser-induced transformation of precursors for realization of extremely localized C-Au-Ag NPs deposition process. It allowed us to create μ-chips promising for detection of ultra-low-volumes and concentrations of bio-agents and hazardous compounds^20^.

It should be noted that photochemistry of relative complexes has not been studied yet from the viewpoint of photo-induced decomposition and associated nano-phase formation. Similar processes are typically considered to be undesirable, especially for the complexes with enhanced luminescence characteristics to keep their useful properties under irradiation. This is why the present study is of critical importance for understanding of laser irradiation effect onto these supramolecules, routes of energy transfer in the complexes of this sort and nature of transformation processes resulting in nanostructured phase formation. Finally this will also make possible a rational design of the hybrid structures with a desired composition, morphology and architecture.

## Results and Discussion

A promising approach to hybrid NPs’ synthesis can be based on transformation of precursor molecules which can be considered as building blocks for construction of final nanostructures. Under the framework of this approach the photosensitive supramolecular complexes, which consist of central heterometallic cluster core (ca. 2 nm size) and surrounded by phosphine and alkyl ligands, can be used as prospective precursors. It should be noted that these compounds may have variable cluster core size, qualitative and quantitative composition (Au−Ag, Au−Cu, Ag−Cu pairs) as well as different organic ligand environment^21^,^22^,^23^ that opens ample opportunities for the NPs’ synthesis with predefined composition, morphology and functional properties.

**Figure 1:**
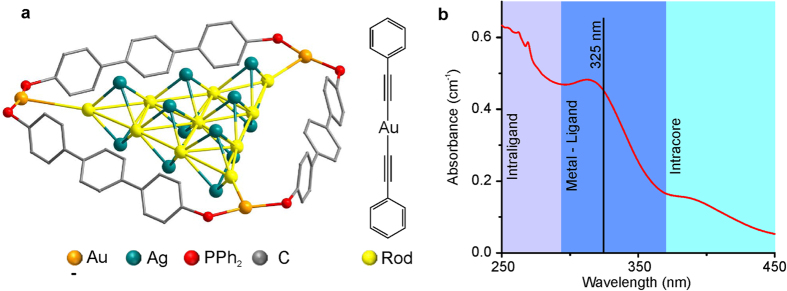
(**a**) Schematic structure of **1**. Hydrogen atoms are omitted for clarity. (**b**) The UV-Vis absorbance spectra of **1**.

Schematic structure of supramolecular precursors [(Au_13_Ag_12_(C_2_Ph)_20_)(PPh_2_(C6H_4_)_3_PPh_2_)_3_](PF_6_)_5_, **1** and [Au_12_Ag_12_(C_2_Ph)_18_X_3_(PPh_2_(C_6_H_4_)_3_PPh_2_)_3_](PF_6_)_3_ (X = Cl, Br, I), **2−4** are shown in Fig. 1(a) and S1 respectively. All these compounds are members of the class ‘rods-in-belt’ clusters that were synthesized and described earlier^22^,^24^. The Au−Ag cluster core of **1−4** is held together by a network of metallophilic interactions that can provide the seeds for formation of the structures with nanoscale characteristic components. The heterometallic cluster core of the supramolecular complex is the chromophoric center, which largely determines the photochemical properties of the complex including its photosensitivity and specific absorbance in the UV spectral region.

Figure 1(b) shows the absorbance spectra of **1** with three main absorption regions^21^,^22^,^24^,^25^: (i) 200–300 nm – high energy absorption due to intraligand (IL) electron transition, (ii) 300–350 nm – absorption due to the metal-to-ligand transition (MLT), and (iii) 370–450 nm – transitions between orbitals of the cluster core (MMT). All these bands can be used for luminescence excitation of the supramolecule complexes, however IL band (200–300 nm) is typically not usable due to high absorbance of majority of components of the system and materials (solvents, reaction vessels, etc.) in this spectral region, MMT band has rather low absorption cross-section that results in low efficiency of energy absorption. This is why radiation of He-Cd laser (325 nm wavelength) was used for optical excitation of **1** into the 300–350 nm MLT absorption band.

**Figure 2:**
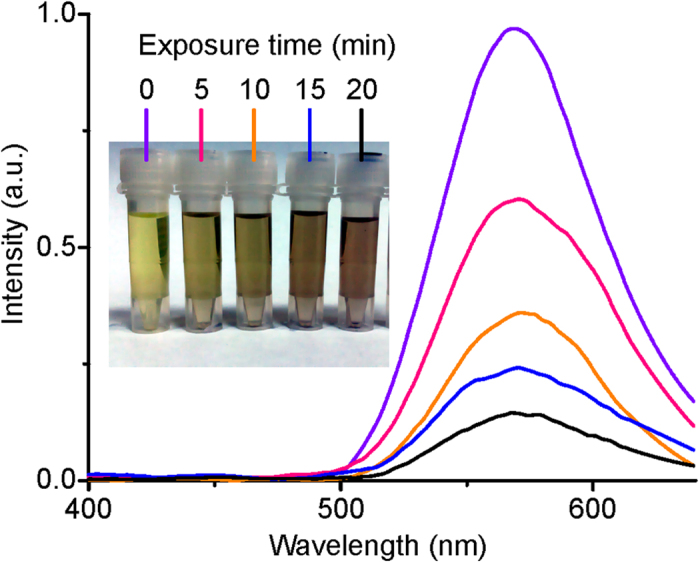
PL intensity and photo of solutions irradiated with He-Cd laser beam.

Laser irradiation of **1** in dichloroethane solution results in intense luminescence (in spectral region 500–700 nm with the maximum at 570 nm), which was found to decrease with irradiation time (Fig. 2). The photoluminescence (PL) intensity was measured for initial solution and solutions irradiated for 5, 10, 15, and 20 min. Along with luminescence fading the solution darkening and opacification (inset Fig. 2) and eventually suspension formation were observed. It should be noted that 60 min irradiation results in complete disappearance of luminescence. The observed effect is evidently indicative of two possible relaxation channels of the complex excited states: “optical channel” – radiative decay to give 570 nm luminescence emission and “transformation channel” – multiphonon relaxation accompanied by transformation/ decomposition of the molecule resulting in a sediment formation.

**Figure 3:**
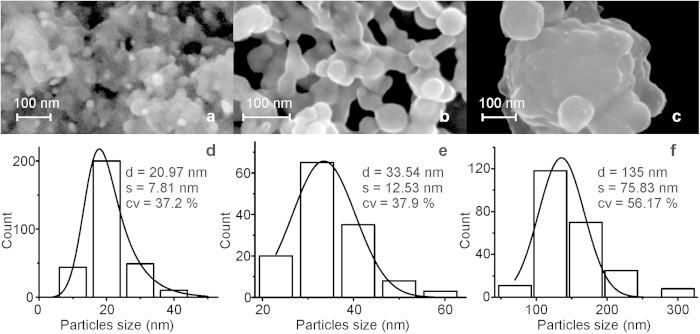
SEM images of NPs obtained from (**a**) acetone, (**b**) acetophenone, (**c**) dichloroethane solutions of **1**. (**d–f**) Size distribution of the NPs.

Prolonged laser beam irradiation of **1** solution allows for formation of the nanoparticles with morphology and size which depend on physicochemical properties of solvent. SEM images of the NPs formed in different solvents (acetophenone, dichloroethane and acetone) and separated from solutions by centrifugation are shown in Fig. 3a–c. The density of solvents used increases in the acetone, acetophenone, dichloroethane sequence (0.79, 1.03, 1.26 g/cm^3^, respectively) and the size of NPs follow the same order that reflects a certain effect of the solvent property. Figure 3d–f presents size distribution of the particles, which was estimated by measuring the particles diameters in the micro-photos. It is clear that the nature of the solvent affects strongly both, the NPs morphology and size. The particles formed in acetone are densely packed and their average dimension is about 20 nm. In acetophenone solution the NPs form a porous structure with the average particle size of 30 nm, whereas large particles with the average dimension ca. 135 nm and rather wide particle-size distribution were found to be formed in dichloroethane solution.

It was found that the concentration of the supramolecular complex affects only the NPs amount without substantial changes in morphology and size of the NPs. The EDX spectrum of the nanoparticles obtained from acetone solution of **1** is presented in Figure S2 (Supporting Information). The data obtained clearly demonstrate that the particles consist of carbon, gold and silver. The component ratio was found to be ca. 90/5/5 at % respectively. It is worth noting that the supramolecular complex **1** used in these experiments consists of 286 atoms of carbon, 13 atoms of gold, and 12 atoms of silver that corresponds to atomic ratio 92/4/4 and evidently predefines the composition of the nanoparticles formed.

**Figure 4:**
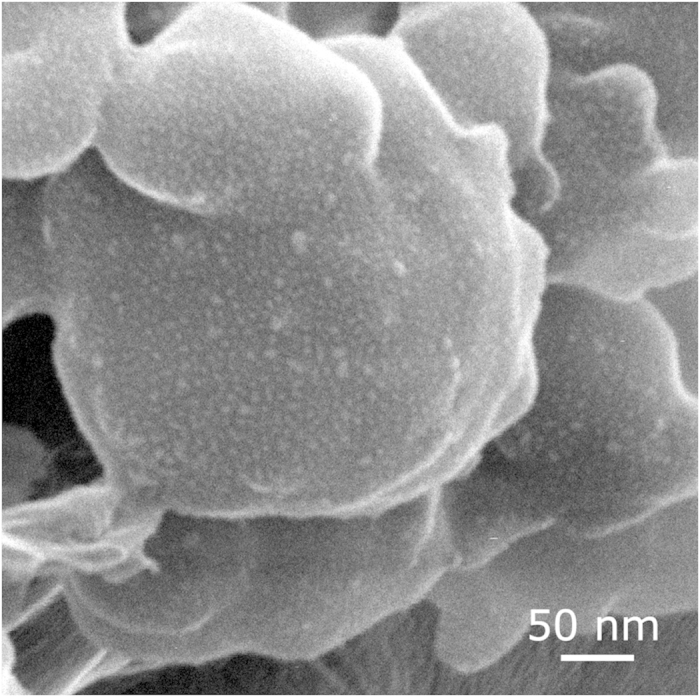
The image of deposited NPs obtained with helium-ion microscope.

High resolution transmission electron microscopy (TEM) image of a single nanoparticle (Figure S3, Supporting Information) demonstrates regions with fringed facet that implies the presence of crystalline patterns in the NPs. Additionally, inhomogeneous contrast of the 30-nm nanoparticle demonstrates that it comprises several sphere-like crystalline structures. The image of large NPs agglomerate obtained with ion microscope is presented in Fig. 4. It is clearly seen that the deposited NPs form inhomogeneous structures which consist of 1–5 nm nanophases located at the distance of ca. 2–5 nm and dispersed in the bigger NPs that points to a composite particles structure – combination of crystalline (Au−Ag) and amorphous (carbon) phases. Bright spots observed in Fig. 4 are the regions which have high secondary electron yield. This fact can be explained in terms of high material and electron density that is typical of metal nanoparticles.

Normalized absorption spectra of nanoparticles obtained from acetophenone, dichloroethane and acetone solutions of **1** are demonstrated in Figure S4 (Supporting Information).

The spectra display a single absorption band in the 400–500 nm range related to the surface plasmon resonance (SPR). The position of the absorption band is typical for the Au−Ag structures and is indicative of the heterometallicity of the crystalline phases formed in the NPs^26^. The observed shift of the plasmon resonance frequency is evidently a result of cooperative effect of the composition and structure of the particles. It should be noted that the SEM, EDX, TEM data and absorption spectra are indicative of complicated inhomogeneous structure of the nanoparticles which consist of 20–100 nm amorphous carbon spheres with encapsulated 1–5 nm crystalline nanoclusters of the Au−Ag alloys.

The presented results were obtained for the nanoparticles synthesized in the bulk solution. It was also found that the laser-induced deposition of NPs is also possible on the surface of the substrate. Figure S5 (Supporting Information) shows the SEM pictures of NPs from different complexes of ‘rods-in-belt’ family on the microscope-cover glass surface. It was found that the NPs composition and morphology are also affected by the substrate material and the type of solvent. The material formed from **1** contains strongly agglomerated NPs with dense morphology. Solutions of complexes **2−4** give more porous NPs layers. Careful examination of the images shown in Figure S5 (Supporting Information) allows to conclude that the morphology (density and porosity) of the NPs obtained also depends on the halide ions nature.

### Raman and FTIR analysis

The EDX spectra revealed that the NPs contain up to 90 at % of carbon. In order to study the allotropic form of the carbon phase, the Raman spectroscopy was applied, which considered to be a sensitive tool for analysis of carbon containing materials. The Raman spectroscopic data may provide information concerning the carbon phase “bonding structure” by determination of the sp^2^ (graphite-like) to sp^3^ (diamond-like) hybridization ratio^27^, which essentially defines their physical properties. It was shown that carbon rich materials are characterized by specific Raman bands in the 800–2000 cm^−1^ region with the so-called G peak at 1560 cm^−1^ and D peak at 1360 cm^−1^. G-peak is typically determined by the bond stretching of all pairs of sp^2^ atoms in rings and chains. The D peak originates from the breathing modes of sp^2^ atoms in rings. The position of the D peak is determined by the concentration of sp^3^ hybridized carbon^28^. The Raman spectra of carbon materials depend on the sp^2^ phase clustering, bond-length and bond-angle disorder, presence of sp^2^ rings or chains, the sp^2^/sp^3^ ratio^29^. In accordance with^30^ intensity ratio of D and G peaks (I(D)/I(G)) allows to conclude on the carbon allotrope and graphite cluster size.

**Figure 5:**
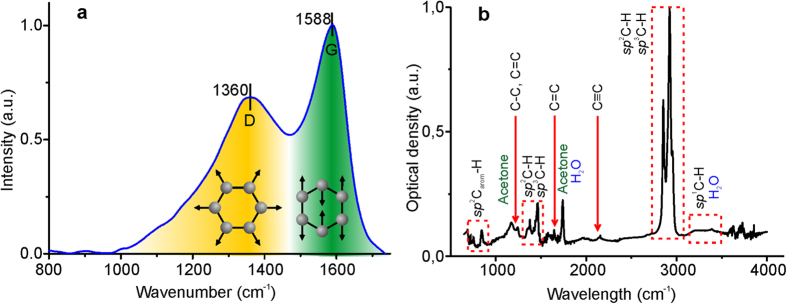
(**a**) Raman spectrum of deposited NPs. (**b**) FTIR spectrum of the Au-Ag@a-C:H hybrid.

The Raman spectrum of the hybrid Au-Ag-C nanoparticles is presented in Fig. 5(a). It displays two bands centered at 1350 and 1587 cm^−1^ associated with D and G component of the carbon Raman signal. In accordance with^31^ the band intensities and positions are typical for the hydrogenated amorphous carbon a-C:H. The I(D)/I(G) ratio was found to be about 0.75 that is indicative^30^ of the a-C:H allotrope formation with 12% of sp^3^ bonds and graphite cluster size about 8–12 Å.

The IR spectroscopy is another method that allows for non-invasive monitoring of the reaction course and material characterization. The FTIR spectra show the time-dependent evolution of the complex **1** under laser irradiation (see Figure S6, Supporting Information). It is worth noting that decomposition of **1** in solution is accompanied by accumulation of а substance with high content of C−H bonds (the signals at 1400 and about 2900 cm^−1^ correspond to oscillations of sp^2^C−H and sp^3^C−H bonds^31^,^32^. Additionally the signals corresponding to the sp^1^C−H, C=C, C≡C, C−C, and sp^2^Caromatic−H bonds oscillations have been found in the fingerprint region of FTIR spectrum of the NPs separated from reaction mixture (the spectrum with the signals assignment is shown in Fig. 5(b)).

The information provided by the Raman and FTIR measurements support the hypothesis that the carbon matrix of the nanostructures under study is either amorphous hydrogenated a-C:H carbon with low hydrogen content (less than 20 at%) or graphite-like a-C:H (GLCH)^31^,^32^,^33^.

### Characterization of hybrid C-Au-Ag Nanoparticles by XPS

In order to characterize the nature and estimate the size of the hybrid Au-Ag@a-C:H nanoparticles we studied Au 4f and Ag 3d photoemission (PE) core-level spectra. It is worth noting that analysis of PE spectra of the hybrid Au−Ag NPs should take into account not only cluster-size effect (i.e. quantum confinement effect)^34^,^35^ but also formation of nanoalloy in the heterometallic species and a possible charge transfer from gold to silver atoms^36^. The former effect appears in the PE spectra as a shifts of core levels to higher binding energies (BEs) with respect to the bulk metal values and tend to increase with the decrease of the NP size. This trend has been reported^31^,^32^,^33^ for the metal nanoclusters of relatively small size, lower than 5–6 nm. Some of the authors explain this behavior in terms of ground state effect caused by sp-d rehybridization (modification of the repulsive interaction between core levels and valence electrons)^35^. Another interpretation of these shifts consists in final state effect due to inability of the system to effectively shields the core hole generated by the photoemission process. Therefore the positive charge of the nanoparticle in the photoionization process results in the loss of photoelectron kinetic energy^34^. Size of the NPs can be estimated by comparison of the values of the BE shift of the corresponding core-level band with the data cited in literature^37^. On the other hand, the charge transfer from Au to Ag atoms, which was previously revealed for the AuAg alloys^38^ and gold–silver–gold double-shell (Au@Ag@Au) NPs^36^, also affects the spectra. Apparently, these processes result in the shift of the Ag 3d bands to lower BE values whereas the Au 4f signals should be shifted towards higher BE^38^,^39^.

**Figure 6:**
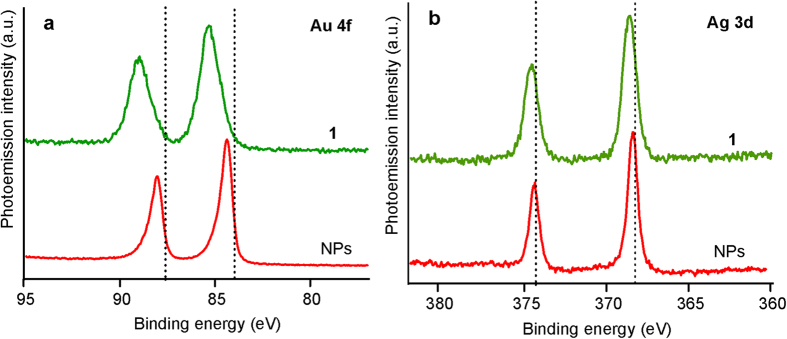
(**a**) Au 4f and (**b**) Ag 3d PE core-level spectra obtained for the Au–Ag NPs (red) and precursor **1** (green).

Figure 6 shows the Au 4f (a) and Ag 3d (b) PE core-level spectra of the hybrid Au-Ag NPs and the precursor complex **1**, dashed lines indicate the positions of the bulk metal bands. The structure of **1** spectra corresponds to those of the Au(I) and Ag(I) compounds as it was discussed previously^25^. For the laser-induced NPs we observe asymmetric Au 4f peaks which are shifted towards higher BE by ~0.4 eV relatively to the bulk Au. The asymmetric band shape tailing to higher energy can be explained by the presence of significant lognormal cluster size-distribution^40^. The evaluation of the Ag 3d spectra of hybrid NPs revealed higher energy BE shift of ca. 0.1 eV relative to the bulk metal value. It is worth mentioning that analysis of the NPs size in terms of the cluster-size effect without consideration of the Au−Ag alloy formation may result in overestimation the NPs size from the Ag 3d core-level BE measurements and, at the same time, underestimation from the Au 4f spectra. For example the values obtained from analysis of the Ag 3d spectrum^37^ amount to ca. 6 nm whereas the corresponding value drops down to ca. 2 nm calculated using the Au 4f BE shift^40^. The real NPs’ mean size seems to be in-between, i.e. 3–5 nm. This estimation is in good agreement with the results obtained by SEM and TEM.

**Figure 7:**
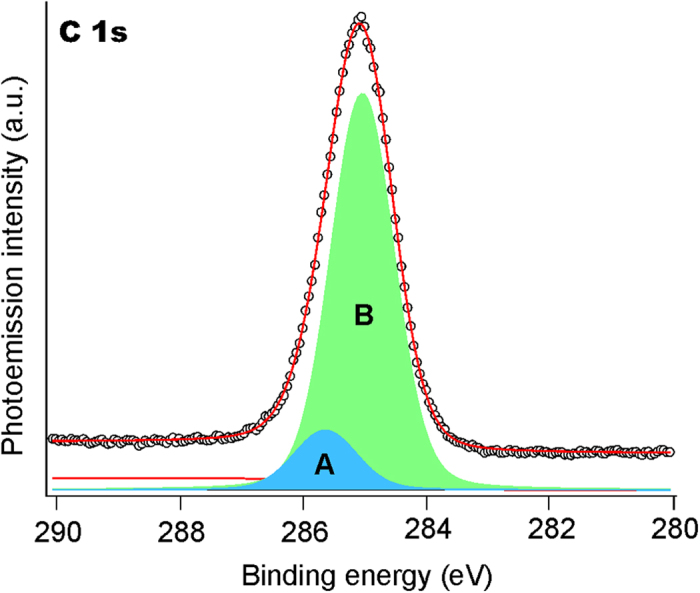
C 1 s core-level spectra for the Au–Ag NPs embedded in carbon matrix.

According to the well-elaborated approach of quantitative X-ray photoelectron spectroscopy (XPS) measurements it is possible to estimate quantitative composition of the NPs obtained. In particular, the Au to Ag ratio proved to be 9:10, whereas analysis of the C 1 s core-level spectra allows revealing the nature of carbon matrix in which the Au−Ag NPs are embedded^41^. The deconvoluted C 1 s spectrum of the AuAg@C NPs is shown in Fig. 7. The band at higher BE (A) may be associated with the sp^3^- hybridized carbon atoms and band B is attributed to sp^2^- hybridized carbons, the amount of the former is about 13%.

In conclusion, the results of absorption, Raman and FTIR spectroscopies, together with the data of XPS, SEM and EDX analysis indicate that the deposited NPs display a complex morphology, which consists of the Au–Ag nanoalloys (nanoclusters) (3–5 nm) separated by the distance of ca. 2–5 nm and incorporated into the carbon matrix with the structure of hydrogenated amorphous carbon a-C:H (Au-Ag@a-C:H NPs). The NPs could be called multi-yolk-shell hybrid nanostructures. It is important to note that the dimension and morphology of Au-Ag@a-C:H NPs can be controlled by variations of the experimental parameters, namely solvent, nature and composition of the rods-in-belt supramolecule precursor, deposition conditions (in bulk solution or onto the surface of the substrate) and duration of laser irradiation.

Functional properties of the obtained nanostructures are determined by their plasmon resonance characteristics (position, half-width at half-maximum) as well as by biocompatibility. The most striking example of plasmon NPs’ properties is the Surface-Enhanced Raman Scattering. The hybrid Au-Ag@a-C:H NPs are the ensembles of bimetallic Au–Ag nanoparticles allocated at the distance 2–3 nm in carbon matrix. Such structures can be considered as agglomerated “hot spots”, which are promising from the viewpoint of SERS sensing for the direct detection of low concentrated species (proteins, viruses, etc.) up to single molecule level that is particularly challenging. To study the availability of the Au-Ag@a-C:H NPs for the SERS analysis we used human serum albumin (HSA) as a test substance, which belongs to the class of proteins with poor detectability. Spatially resolved Raman spectra for 0.1 μl drop of HSA water solution (concentration 10^−6^ M) onto substrate with Au-Ag@a-C:H NPs are presented in Figure S7 (Supporting Information). Line scan across agglomerate of HSA molecules demonstrates prospectivity of Au-Ag@a-C:H nanoparticles for the SERS label-free detection. The obtained results make Au-Ag@a-C:H NPs extremely promising for medical application and clinical diagnosis, for example for investigation of protein-rich biofluids and cancer cell detection^42^.

### Au-Ag@a-C:H NPs formation mechanism

A series of experiments described above demonstrated that laser irradiation of solutions of the supramolecular complex results in decomposition of the starting compound to form the hybrid Au-Ag@a-C:H NPs. Depending on laser radiation intensity the process of NPs formation can be either photo- or thermally initiated. A conclusion on the nature of laser-induced transformation can be done from the estimation of the temperature in the laser focal spot. Figure S8 (Supporting Information) presents the spatial temperature distribution profiles obtained using heat conductivity equation. For the laser intensity used in these experiments (0.1 W/cm^2^) the maximum temperature in the laser focal spot does not exceed 25 °C that testifies in favor of photo-induced initiation of the hybrid NPs formation.

**Figure 8:**
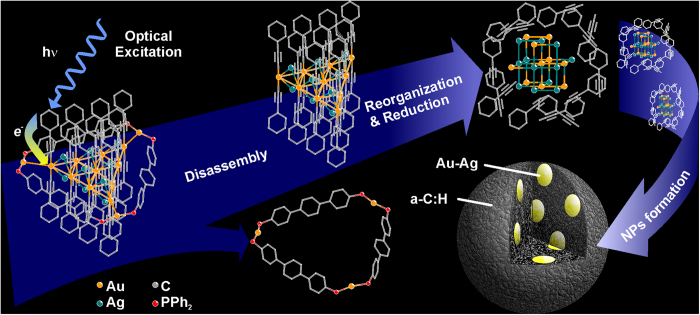
Schematic presentation of Au-Ag@a-C:H NPs formation mechanism.

The following scheme of the NPs formation can be suggested on basis of the experimental data available. At the first stage, the complex absorbs 325 nm laser irradiation due to MLCT transition. It was found that the complex excitation into intraligand electron transition (250 nm) does not cause NPs formation. The photo-excitation of the molecule through MLCT results in the rupture of metallophilic bonds between central [Au_10_Ag_12_(C_2_Ph)_20_]^2+^ cluster core and [Au_3_(PP)_3_]^3+^ “belt” to leave in solution two independent species (Fig. 8). The latter species remains in solution and is not involved in the formation of NPs. The mass spectroscopic study of the resulting reaction mixture provides a strong support for the hypothesis given above. The ESI + mass spectrum of the final solution obtained after NPs centrifugation displays two dominant signals at 796 and 944 m/z (Figure S9, Supporting Information). The former is a combination of the signals generated by two independent species [Au_3_(PPh_2_(C_6_H_4_)_3_PPh_2_)_3_]^3+^ and [Au_2_(PPh_2_(C_6_H_4_)_3_PPh_2_)_2_]^2+^ derived from the gold-diphosphine “belt”, note that the dimer is thermodynamically stable compound isolated and characterized earlier (Figure S10, S11, Supporting Information)^21^. Another intense signal at 944 m/z corresponds to the product of partial recombination ([Au_2_(PPh_2_(C_6_H_4_)_3_PPh_2_)_2_(AuC_2_Ph)]^2+^, Figure S12, Supporting Information) of the reaction mixture components. The position of the signals and their isotopic distribution fit completely the composition given above (Figure S11, S13, Supporting Information).

In the next step the {Au_10_Ag_12_(C_2_Ph)_20_}^2+^ species (cluster core of the initial complex) lose chemical stability and undergoes a complex chemical transformation. This transformation includes several stages such as breaking off phenyl radicals from ‘rods’, formation of hydrogenated carbon matrix, and the simultaneous reduction and aggregation of the metals (Fig. 8). The transformation mentioned above evidently includes several successive stages at least one of which can be a photochemical process. The observation that the Au/Ag ratio of the cluster core in the starting compound matches well the composition of encapsulated heterometallic species in the NPs isolated from reaction mixture is another indication in favor of the mechanistic model suggested.

## Conclusions

The present study revealed that laser irradiation of solutions of the supramolecular [{Au_13_Ag_12_(C_2_Ph)_20_}(PPh_2_(C_6_H_4_)_3_PPh_2_)_3_](PF_6_)_5_ and [Au_12_Ag_12_(C_2_Ph)_18_X_3_(PPh_2_(C_6_H_4_)_3_PPh_2_)_3_](PF_6_)_3_ (X = Cl, Br, I) complexes results in formation of the hybrid Au-Ag@a-C:H NPs with the components ratio (C/Au/Ag) matching well the composition of the starting precursor. Variation of the solvent, supramolecular precursor and irradiation parameters allows for changing the NPs size and morphology. The obtained NPs can be used for SERS experiments and detection/identification of low concentration of bioorganic analytes. The photochemical formation of the Au-Ag@a-C:H NPs can be considered as an efficient and promising approach to the synthesis of multifunctional nanostructures. The most attractive feature of this approach is fine tuning of morphology and properties of the NPs through variations of easily controlled technological parameters of the process (composition of supramolecule precursor, solvent properties, reagent concentration, irradiation time, and deposition geometry). The mechanism of hybrid Au-Ag@a-C:H NPs formation was found to be photoinduced decomposition of the precursor molecule followed by transformation of the cluster core (red-ox intramolecular reaction and aggregation of heterometallic species) to give the hybrid metal/carbon nanostructures with multi-yolk-shell architecture. In this case the composition of the complex cluster core predefines the composition of the reduced heterometallic species, the carbon-rich organic ligand provides building blocks for carbonaceous matrix, whereas the deposition parameters dictate morphology of the NPs formed. The other ways of preparation of analogous metal-carbon structures suggested earlier is a sophisticated multistage organometallic chemistry, which appears to be even more complicated in the synthesis of hybrid multi-metallic nanoscale materials. Thus the laser-induced transformation of supramolecular precursors resulting in formation of hybrid bimetallic nanoparticles is promising strategy for preparation of materials of this type.

## Methods

### Solution preparation

The supramolecular complexes used [{Au_13_Ag_12_(C_2_Ph)_20_}(PPh_2_(C_6_H_4_)_3_PPh_2_)_3_][PF_6_]_5_ (**1**), [Au_12_Ag_12_(C_2_Ph)_18_Cl_3_(PPh_2_(C_6_H_4_)_3_PPh_2_)_3_](PF_6_)_3_ (**2**), [Au_12_Ag_12_(C_2_Ph)_18_Br_3_(PPh_2_(C_6_H_4_)_3_PPh_2_)_3_](PF_6_)_3_ (**3**), [Au_12_Ag_12_(C_2_Ph)_18_I_3_(PPh_2_(C_6_H_4_)_3_PPh_2_)_3_](PF_6_)_3_ (**4**) were synthesized according to published procedure^22^,^27^. The solutions for the NPs synthesis were prepared by dissolving of 5 mg of the supramolecule complex (**1–4**) in 1 ml of solvent (acetone, dichloroethane and acetophenon of analytically grade purity). Using the lower and higher concentrations affects only on the amount of the NPs obtained, fundamental differences in structure and morphology were not observed. To remove the undissolved components of the complex, the solution was centrifugated at 10000 rpm for 5 min. Then an aliquot of the solution was placed into a quarts cuvette and illuminated with laser beam.

### NPs preparation

Au-Ag@a-C:H nanoparticles were prepared from solution of supramolecule complex by laser-induced liquid phase deposition method^43^. Unfocused beam of the He-Cd laser (CW, λ = 325 nm, I = 0.1 W/cm^2^) was used to irradiate the solution, the diameter of laser spot was ca. 3 mm. The choice of He-Cd laser was determined by the absorption band at 328 nm of the used supramolecular complex. Low laser intensity and unfocused laser beam were used for elimination of thermally induced processes. Photodecomposition of the complex occurred both on the substrate (microscope-cover glass) and in solution that results in formation of a deposit on the surface and NPs suspension in solution correspondingly. NPs formed in the bulk of solution after irradiation were removed from the solution by centrifugation at 10000 rot/min for 10 min and then washed with acetone.

Deposition on the substrate was carried out by the unfocused laser radiation of the substrate-solution interface. Microscope-cover glass substrates were cleaned sequentially with acetone and isopropanol under sonication for 10 min and drying under N_2_ stream. After completion of the deposition process the substrates with precipitated particles were washed with acetone and dried at 40 °C for 50 min.

### Material Characterizations

A scanning electron microscope Zeiss Merlin was used for all SEM based characterization with 10 kV used for imaging and 15 kV used for EDX. A transmission electron microscope Zeiss Libra 200FE was used at 200 kV for all TEM measurements. Measurement of the absorption spectra of solutions and produced nanoparticles performed using a double-beam spectrophotometer Perkin Elmer Lambda 1050. Luminescence spectra were recorded with a Modular Spectrofluorimeter Fluorolog-3, excitation wavelength of supramolecular complex luminescence was 325 nm. Fourier Transform Infrared Spectroscopy (FTIR) analyses were performed on a FT-IR spectrometer Nicolet 8700 (Thermo Scientific) spectrometer equipped with a MCT-A detector. The spectra were taken with a 2 cm^−1^ resolution in a wavenumber range from 4000 to 400 cm^−1^. FTIR analysis was recorded directly dropping the NPs sample in the ATR (diamond crystal) instrument. The SERS measurements were performed under ambient conditions using a Raman spectrometer Senterra equipped with confocal microscope. The SERS spectra were recorded at room temperature using solid state laser λ_ex_ = 532 nm, power 0.2 mW. The laser was focused onto an approximately 10^−6^ cm^2^ spot of the sample surface. The spectra were collected over ten seconds in back reflection geometry through a 50Х0.7NA objective, the reflected signal was focused onto a thermo-electrically cooled CCD array. The spatially resolved Raman mapping was obtained with step 0.25 μm in X and Y directions. All XPS measurements were performed at the Berliner Elektronenspeicherring für Synchrotron Strahlung (BESSY II) using radiation from the Russian-German beamline^44^. Photoemission (PE) spectra were acquired with a hemispherical Phoibos 150 electron energy analyzer (Specs GmbH). Mass spectra were determined with a Bruker maXis HD ESI-QTOF instruments in the ESI^+^ mode.

## Additional Information

**How to cite this article**: Manshina, A. A. *et al.* Laser-induced transformation of supramolecular complexes: approach to controlled formation of hybrid multi-yolk-shell Au-Ag@a-C:H nanostructures. *Sci. Rep.* **5**, 12027; doi: 10.1038/srep12027 (2015).

## Supplementary Material

Supporting Information
