# Supplementary material for: Laser-induced transformation of supramolecular complexes: approach to controlled formation of hybrid multi-yolk-shell Au-Ag@a-C:H nanostructures
Source: Sci Rep. 2015 Jul 8;5:12027. doi: 10.1038/srep12027 (PMC4495562; doi:10.1038/srep12027)
Supplement: Supporting Information [file srep12027-s1.pdf]

Laser-induced transformation of supramolecular complexes: approach to controlled formation of hybrid multi-yolk-shell Au-Ag@a-C:H nanostructures for stable SERS substrates

A. A. Manshina,[a*] E. V. Grachova,[a*] A. V. Povolotskiy,[a] A. V. Povolotckaia,[b] Y. V. Petrov,[c] I. O. Koshevoy,[d] A. A. Makarova,[e] D. V. Vyalikh[e] and S. P. Tunik[a]

[a] *Institute of Chemistry, St. Petersburg State University, Universitetskii pr. 26, St. Petersburg, 198504, Russia.*

[b] *Center for optical and laser materials research, Research park, St. Petersburg State University, Ulianovskaya St. 5, St. Petersburg, 198504, Russia.*

[c] *Interdisciplinary Resource Center for Nanotechnology, St. Petersburg State University, Ulianovskaya St. 1, St. Pe-tersburg, 198504, Russia.*

[d] *University of Eastern Finland, Joensuu, 80101, Finland.*

[e] *Institut für Festkörperphysik, Technische Universität Dresden, D-01062 Dresden, Germany.*

E-mail: bird231102@mail.ru; manshina@chem.spbu.ru

Supporting information

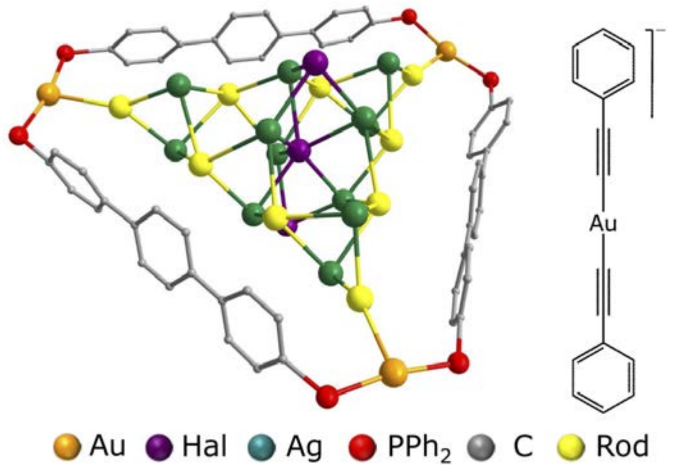

Figure S1. Schematic presentation of the cations **2−4** structure. Hydrogen atoms are omitted for clarity. Hal = Cl, Br, I.

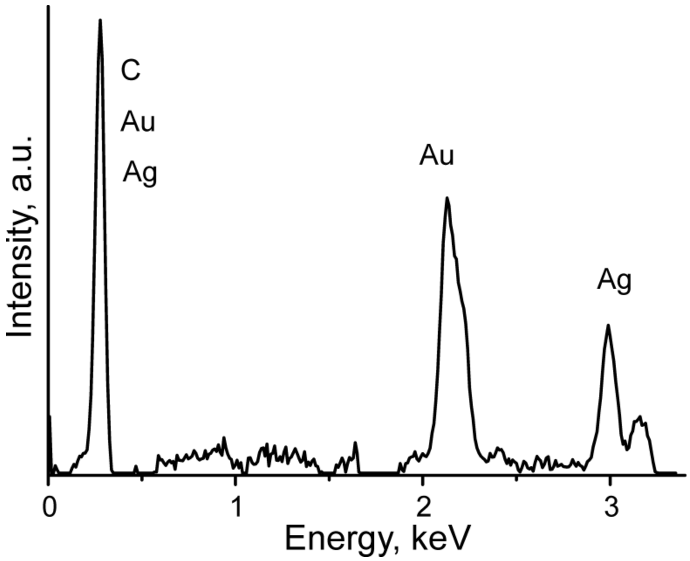

Figure S2. The EDX spectrum of the bulk NPs obtained from acetone solution of **1**.

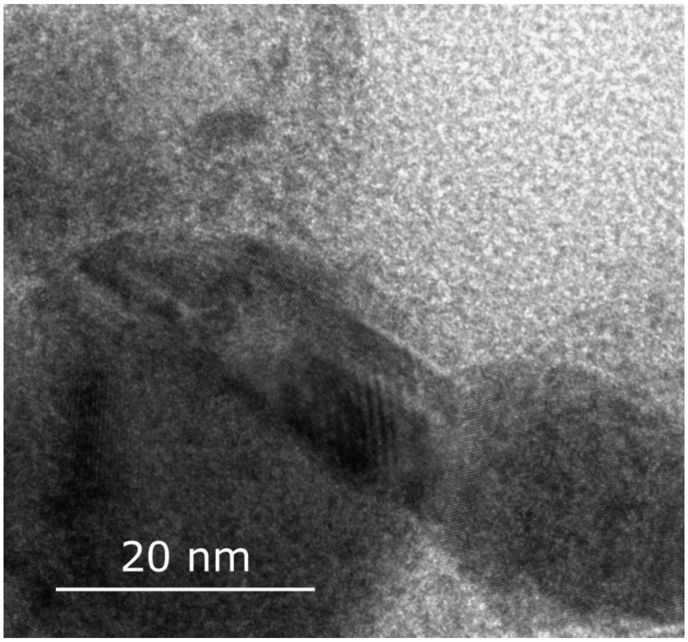

Figure S3. TEM image of NPs deposited from acetophenone solution of **1**.

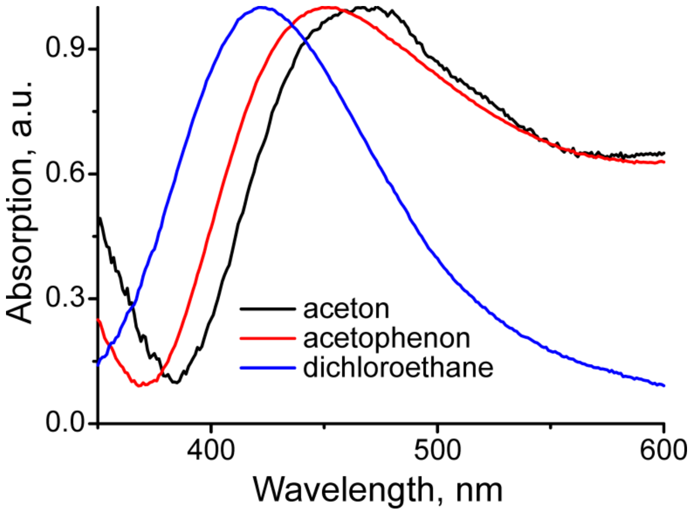

Figure S4. Absorption spectra of NPs from acetophenone, dichloroethane and acetone solutions of the compound **1**.

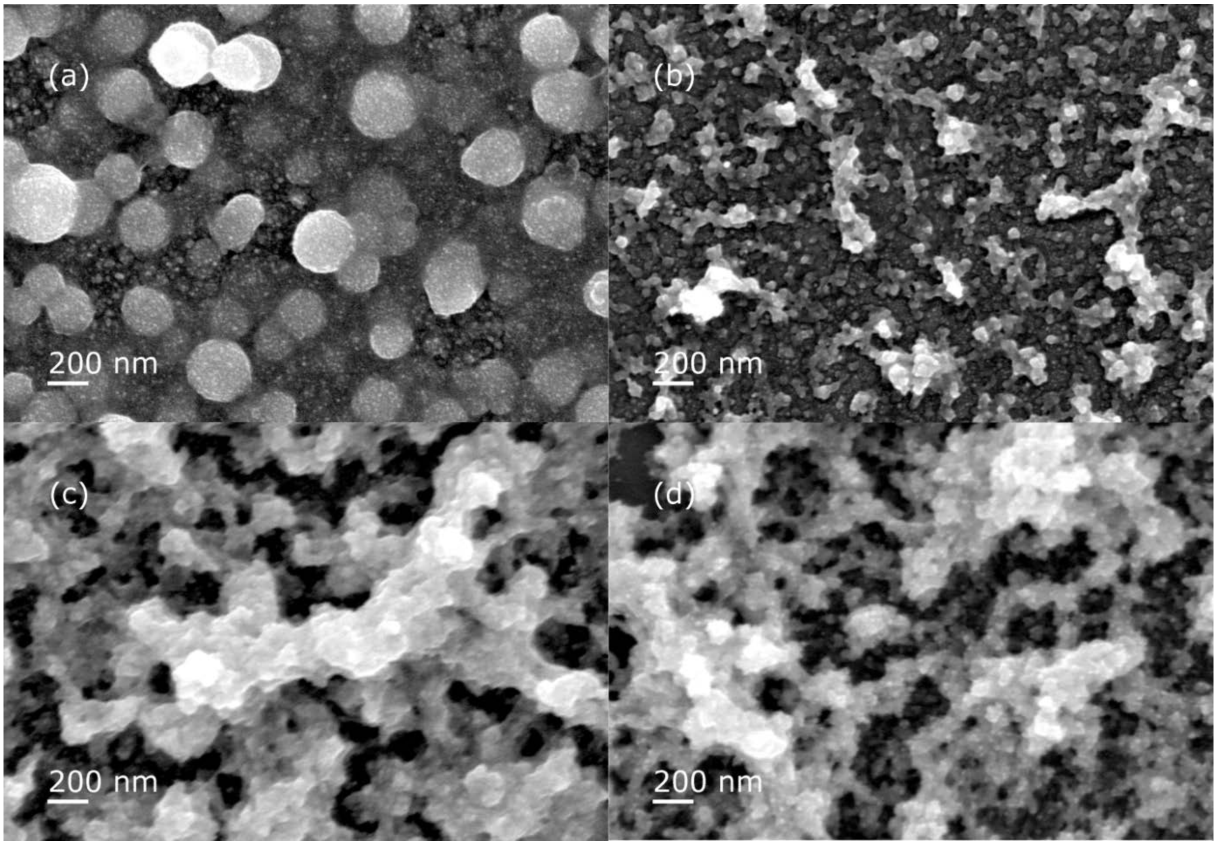

Figure S5. SEM images of NPs obtained from dichloroethane solutions of the complexes **1-4**: (a)-(d) respectively.

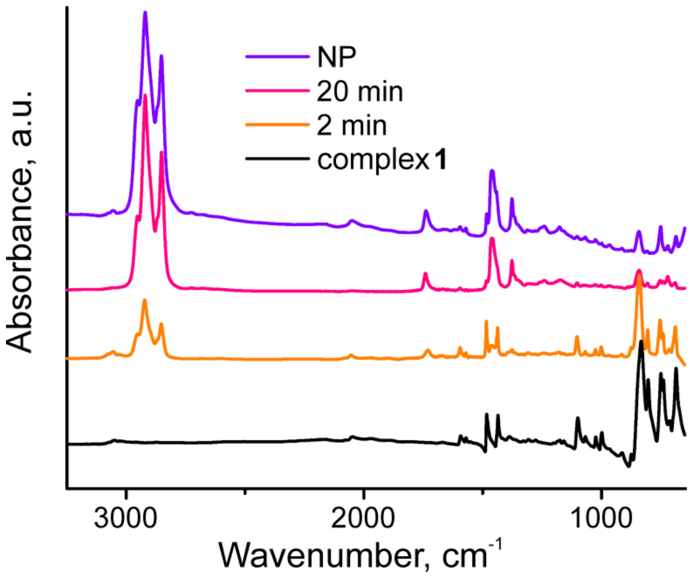

Figure S6. Time-dependent evolution of the complex **1** solution in dichloroethane solution under laser irradiation by FTIR.

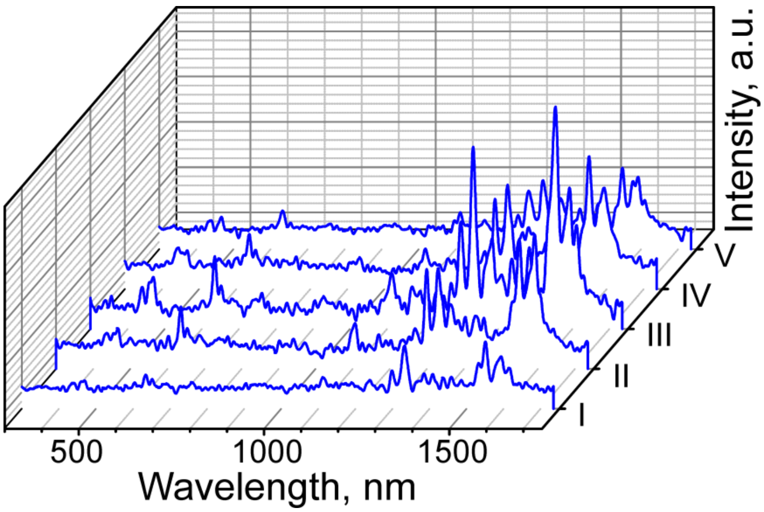

Figure S7. Raman spectra measured from a line scan (I-V points with 0.25 μm distance) across agglomerate of HSA molecules on the substrate with Au-Ag@a-C:H NPs.

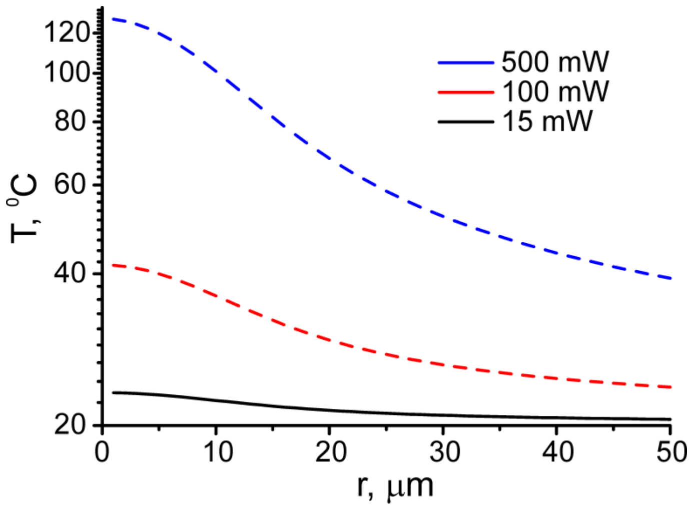

Figure S8. Spatial temperature distribution profiles in the laser affected area.

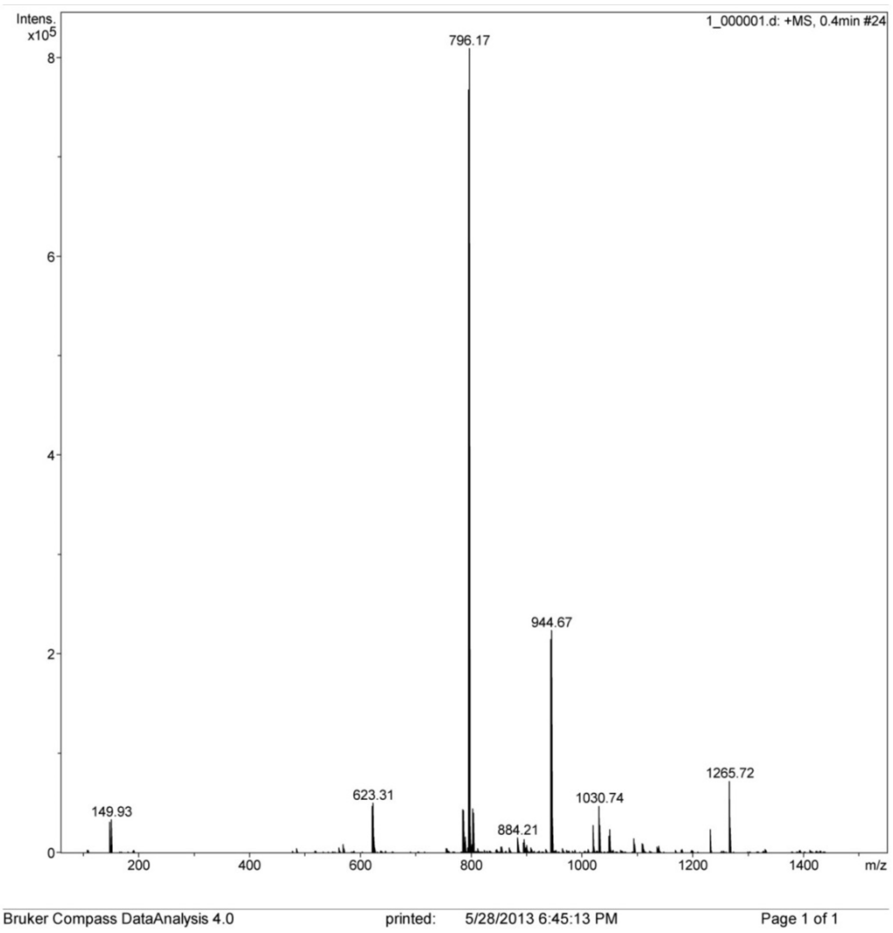

Figure S9. ESI$^+$ spectrum of the compound **1** solution after NPs deposition.

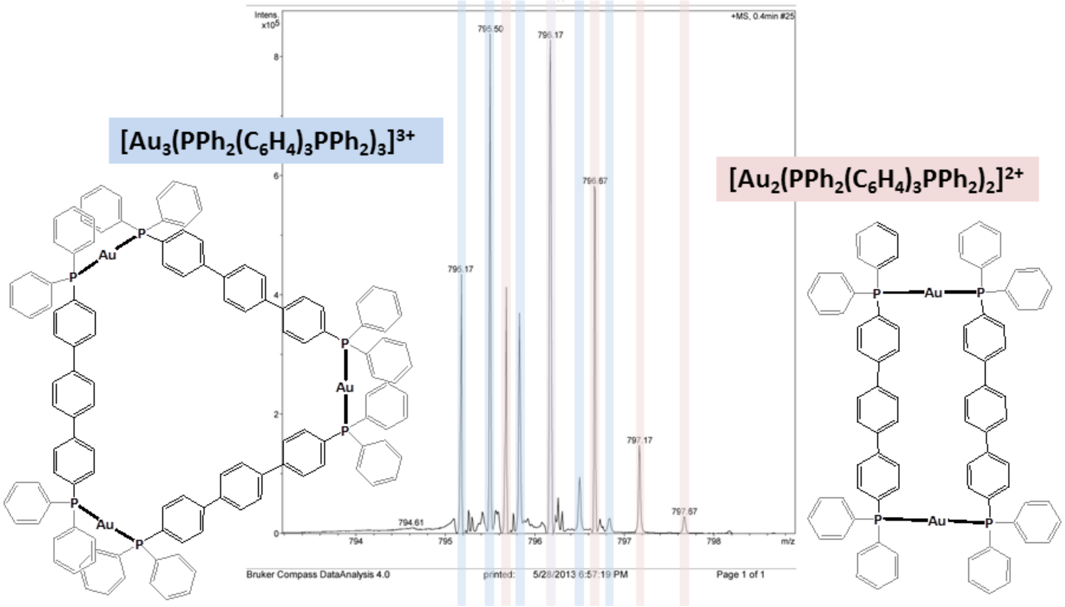

Figure S10. Detailed view of ESI$^+$ spectrum of the compound **1** solution after NPs deposition in the range 793–798 m/z.

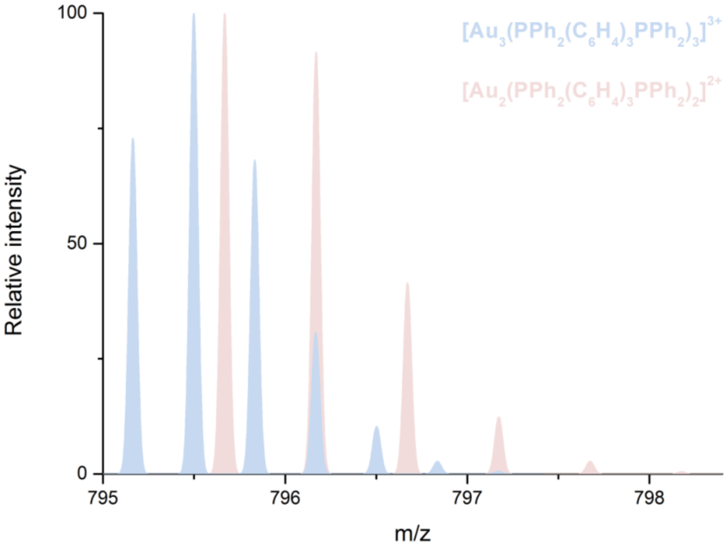

Figure S11. Simulation of molecular ion isotope patterns for two independent species $[Au_3(PPh_2(C_6H_4)_3PPh_2)_3]^{3+}$ and $[Au_2(PPh_2(C_6H_4)_3PPh_2)_3]^{2+}$.

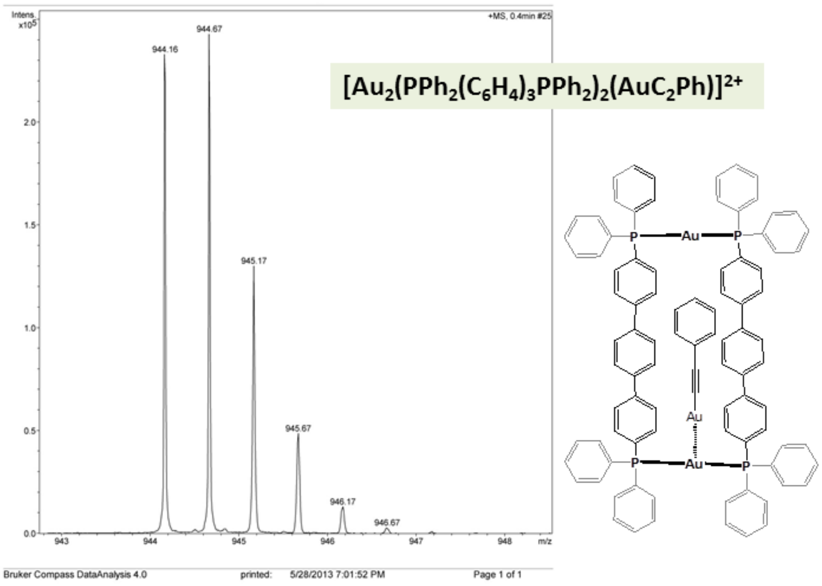

Figure S12. Detailed view of ESI^{+} spectrum of the compound **1** solution after NPs deposition in the range 943–948 m/z.

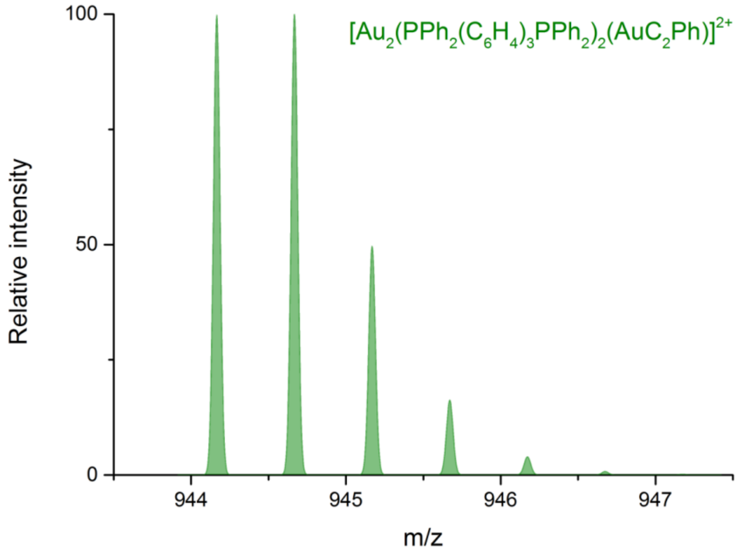

Figure S13. Simulation of molecular ion isotope patterns for $[Au_2(PPh_2(C_6H_4)_3PPh_2)_2(AuC_2Ph)]^{2+}$.
